# Fixed Thoracic Lead Tags Are Associated with Distinct Landing-Approach Trajectories in Honey Bees (*Apis mellifera*)

**DOI:** 10.3390/ani16193152

**Published:** 2026-10-08

**Authors:** Yu-Zhu He, Hui-Dan Ouyang, Chang-Long Li, Ting-Song Lu, Li-Hui Wu, Hong-Ping Wu, Xun Zhong, Jin-Wu Zhang, Xu-Jiang He

**Affiliations:** 1 Honeybee Research Institute, Jiangxi Agricultural University, Nanchang 330045, China; heyuzhu@stu.jxau.edu.cn (Y.-Z.H.); 13694833126@163.com (C.-L.L.); 19100262275@163.com (J.-W.Z.); 2 Vocational Teachers College, Jiangxi Agricultural University, Nanchang 330045, China; ouyanghuidan@jxau.edu.cn (H.-D.O.); wuhongping2022@163.com (H.-P.W.); 19970039940@163.com (X.Z.); 3 School of Computing and Artificial Intelligence, Jiangxi University of Finance and Economics, Nanchang 330013, China; 2202410091@stu.jxufe.edu.cn (T.-S.L.); wlh19170216383@163.com (L.-H.W.)

**Keywords:** *Apis mellifera*, external load, landing behavior, three-dimensional trajectory, flight velocity, trajectory curvature, insect flight

## Abstract

Load carriage is common during honey bee foraging, but its effects on the final approach to the hive remain unclear. We recorded one three-dimensional landing trajectory from each of 80 returning *Apis mellifera* foragers carrying fixed lead tags of 0, 20, 40, or 60 mg attached externally to the dorsal thorax (*n* = 21, 22, 16, and 21, respectively) using three high-speed cameras. We calculated a discrete estimate of trajectory curvature, flight velocity, and vertical velocity. The 20, 40, and 60 mg loads represented approximately 25.69%, 51.37%, and 77.06% of the mean body mass of a separate reference sample (*n* = 60), respectively. Control and 20 mg honey bees generally flew more slowly along more curved paths than 40 and 60 mg honey bees. The 40 mg group had the highest average velocity and lowest average curvature. In the 60 mg group, velocity and curvature varied among recording zones. These results show that landing trajectories differed among load treatments. However, the experimental loads differed from natural nectar or pollen loads, and without measurements of wing kinematics or flight forces, the observed trajectory differences do not identify a specific flight-control mechanism.

## Simple Summary

Foraging honey bees routinely return to their colonies while carrying pollen, nectar, or other materials. Added mass may make it more difficult to slow down, turn, and land at the hive entrance. To examine how a controlled external load is associated with the final approach to the hive, we recorded one three-dimensional landing trajectory from each of 80 foragers carrying fixed lead tags attached externally to the thorax (0, 20, 40, or 60 mg). Honey bees with lower loads generally approached more slowly along more curved paths, whereas honey bees carrying 40 mg flew faster along straighter paths. Honey bees carrying 60 mg showed different changes in speed and curvature across the upper, middle, and lower recording zones. These observations show that attached loads were related to the way honey bees approached the colony and provide a basis for further studies of loaded insect flight.

## 1. Introduction

Foragers return to the colony with nectar, pollen, water, or resin. Nectar is carried in the distensible crop [1], so the mass supported in flight can vary among foraging trips. Additional mass increases the force required to support body weight and can alter momentum, deceleration and turning. These effects may be especially important near a hive entrance, where a returning honey bee must control its speed and path within a restricted space.

Honey bees meet changing force demands by adjusting stroke amplitude and other aspects of wing kinematics [2,3]. In hovering flight, load type and load mass affect metabolic rate and mechanical power [4]. Force production relies on unsteady aerodynamic mechanisms [5,6], while flight capacity also varies with oxygen availability and air density [7,8]. In nectar-loaded honey bees, temperature is associated with changes in wingbeat frequency, stroke amplitude, water balance, and metabolic heat production [9,10]. Work on bumblebees shows that the consequences of loading also depend on load position, body size, wing condition, and environmental clutter [11,12,13,14]. Thus, the effect of load should be evaluated in the specific behavioral context in which the animal flies.

Landing is a visually guided behavior that requires coordinated deceleration and path control [15]. Honey bees use optic flow and image-expansion cues to regulate approach speed [16,17,18], and their landing maneuvers vary with surface orientation and the stage immediately preceding contact [19,20,21]. During turning near a blocked nest entrance, speed and path curvature are also coordinated [22]. Gaze, the angular extent and contrast of visual patterns, airflow, wind, and moving obstacles can further alter flight or antennal responses [23,24,25,26,27,28]. These studies show that landing trajectories respond to visual, mechanical and environmental conditions. However, the relationship between a fixed external load and the final three-dimensional approach of a forager returning to its colony remains insufficiently described.

Natural foraging loads and the experimental loads used here differ in their physical configuration. Nectar is carried internally in the crop, whereas pollen is carried externally on the hind legs [4]. In contrast, the lead tags used in the present study were fixed externally to the dorsal thorax. These loading configurations differ in position and distribution and may therefore affect the center of mass, moment of inertia, and aerodynamic profile differently [4,11]. The thoracic lead tags should thus be regarded as a controlled experimental loading condition rather than as a direct simulation of natural nectar or pollen carriage.

The present study examined whether fixed external thoracic lead tag treatments were associated with differences in the three-dimensional landing trajectories of returning honey bee foragers. We compared flight velocity, vertical velocity, and trajectory curvature among honey bees assigned to 0, 20, 40, or 60 mg treatments and examined whether these variables differed among the upper, middle, and lower zones of the recording volume. Based on the increased mechanical demands associated with added mass, we expected trajectory measures to differ among treatments, particularly under the higher-load conditions and near the final stage of the recorded approach. The study focused on recorded trajectory characteristics and did not test a specific aerodynamic, sensory, or neural control mechanism.

## 2. Materials and Methods

### 2.1. Behavioral Experiment

Behavioral observations were conducted with three healthy, ten-frame western honey bee colonies (*Apis mellifera*) maintained at the Honeybee Research Institute of Jiangxi Agricultural University in Nanchang city, Jiangxi province, China (28°45′58.05″ N, 115°49′45.79″ E; altitude: 53 m). The independent body-mass reference sample was collected at the same apiary and during a comparable season and sampling period, although the colonies had been replaced. The Institutional Animal Care and Use Committee of Jiangxi Agricultural University, China, approved all experimental procedures using honey bees in our study with an approval number of jxaull-2021-25. All experiments were conducted during the spring season on sunny and warm days. Returning workers carrying pollen pellets on their hind legs were identified as foragers and captured at the hive entrance. The pollen pellets were removed before treatment. Observations of returning marked foragers were conducted between 12:00 and 17:00. The mean air temperature, temperature range, and mean wind speed during each observation period are summarized in Supplementary Table S7.

**Figure 1:**
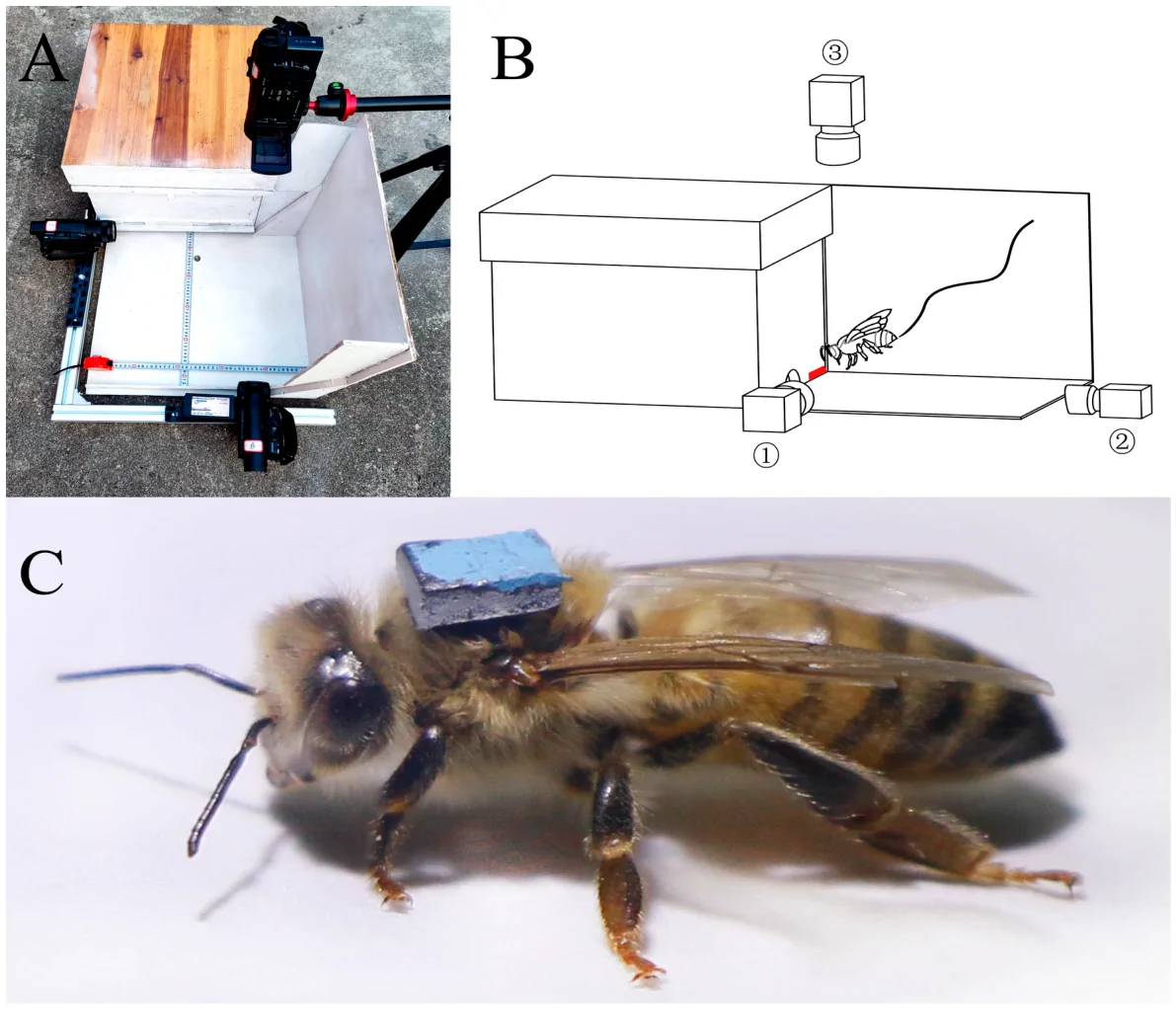
Experimental arrangement. (**A**) The experimental hive and 3D camera recording system. (**B**) The diagram of the setup. Cameras 1, 2, and 3 recorded the xz, yz, and xy projections of the recording volume, respectively. The xz and yz views yielded the x-, y-, and z-coordinate information, while the xy view provided complementary horizontal (x-y) information. (**C**) Forager with a lead tag attached to the thorax. The red box is the entrance to the hive.

Previous measurements indicate that foraging honey bees can carry substantial pollen and nectar loads [4], demonstrating a considerable carrying capacity. We therefore used square lead tags with nominal masses of 20, 40, and 60 mg (mass tolerance: ±0.5 mg) to impose fixed external loads. Each tag had a side length of approximately 2.5 mm, and the three load treatments had approximately the same nominal length and width and therefore approximately the same nominal planar surface area; differences in mass were produced by differences in tag thickness. Each tag was attached with gel super glue (Ningbo Hopson Chemical Industry Co., Ltd., Ningbo, China) to the dorsal thoracic surface at approximately the same position among individuals (Figure 1C). Adhesive-based attachment of small external tags to the dorsal thorax has been widely used in honey bee behavioral studies [29,30,31,32]. The attachment position was kept as consistent as practicable to reduce variation in load placement among honey bees. Control foragers had their hind-leg pollen pellets removed and received both color marking and a sham application of adhesive, but no lead tag. Because wing kinematics and aerodynamic forces were not measured, the experiment could not independently quantify possible effects of the externally attached tags on wing movement or aerodynamic drag. The treatments should therefore be interpreted as fixed externally attached thoracic loads rather than as added mass alone. Different colors identified the treatment groups. The marked foragers were released at a site approximately 10 m in straight-line distance from the hive, outside the wall of the courtyard containing the apiary. After leaving the release area, the honey bees did not necessarily return directly to the hive. The present study focused on the final landing approach of honey bees carrying different fixed, externally attached thoracic lead tags as they returned to the hive. The final dataset comprised 21 control honey bees, 22 honey bees in the 20 mg treatment, 16 honey bees in the 40 mg treatment, and 21 honey bees in the 60 mg treatment (total *n* = 80). These values represent the numbers of complete landing trajectories available for analysis.

The experiment evaluated how fixed external loads affected the final landing approach of returning foragers captured in their natural foraging state. After treatment and release, the honey bees returned to the hive freely, and their flight trajectories were analyzed near the hive entrance. Honey bees were not starved or provided with supplemental sugar solution, so their energetic state, crop contents, and motivation to return were disturbed as little as possible. Three colonies were used, and all four load treatments (0, 20, 40, and 60 mg) were applied within each colony. Within each colony, eight consecutively captured pollen-bearing returning foragers formed one sampling block. After removal of the hind-leg pollen pellets, the eight honey bees within each block were allocated to the four treatments, with two honey bees assigned to each treatment. Individuals were selected without intentional selection based on body size, activity, or other observable characteristics. No formal randomization sequence was used for treatment allocation. All four treatments were represented within the same local sampling period.

Because the field experiments were conducted several years ago and the original records were not designed to retain detailed block-level allocation and disposition information, the exact number of sampling blocks completed for each colony and the treatment-specific numbers of honey bees initially assigned before video recording and trajectory selection are no longer available. These quantities therefore cannot be reliably reconstructed retrospectively and were not inferred from the final sample sizes. The available sample sizes represent only honey bees for which complete videos and normal landing trajectories were retained for analysis: control, *n* = 21; 20 mg, *n* = 22; 40 mg, *n* = 16; and 60 mg, *n* = 21. Each honey bee was recorded during a single post-treatment landing approach under its assigned load condition and therefore contributed one complete flight trajectory to the dataset. No individual honey bee was recorded in repeated landing trials. Individual body mass was not measured in the main experiment, and the attached loads were therefore defined by absolute mass rather than individual relative load.

External body-mass reference sample. Individual body mass was not measured in the main experiment. Therefore, the body-mass measurements from this independent reference sample were used only to provide approximate population-level context for the magnitude of the 20, 40, and 60 mg treatments and were not used to calculate individual relative loads for the experimental honey bees. After the original experimental colonies had been replaced, a separate reference sample was collected from three healthy, ten-frame colonies at the same location and during the same season. Twenty returning pollen-bearing foragers were collected from each colony (total *n* = 60). Their hind-leg pollen pellets were removed before weighing them. Individual measurements and colony-specific summary statistics are provided in Supplementary Table S6.

Three digital cameras (Sony FDR-AX700, Sony Corporation, Tokyo, Japan) were positioned around the hive entrance to record landing flights from three viewing directions (Figure 1A,B). Camera 1 recorded the xz projection, Camera 2 the yz projection, and Camera 3 the xy projection of the recording volume. Together, the xz and yz views provided x-, y-, and z-coordinate information, while the xy view provided additional complementary information for the horizontal x- and y-coordinate components. All three cameras were operated at 500 frames s^−1^, and recording was initiated using a single remote-control command. Representative recordings of the landing approaches for the four external-load treatments are provided in Supplementary Videos S1–S4.

During data processing, the recording endpoints were used as the operational temporal reference, and corresponding frames were matched backward across the three camera views. This alignment procedure was applied consistently to all analyzed trajectories. No independent timing marker was recorded during the experiment; therefore, residual inter-camera timing offsets could not be quantified. The same focal honey bee was identified across the camera views by following its spatial position and continuous movement in the corresponding image sequences.

Three fixed landmarks on the hive, each visible in all camera views, were identified manually and used to align the coordinate origins and axis directions of the three views. Two 50-cm rulers graduated at 1 mm were placed perpendicular to one another on the baseboard and served as spatial references. On the reference ruler, 10 cm corresponded to 544 pixels (1680 − 1136 pixels), yielding a conversion factor of 0.01838 cm pixel^−1^. Image distances were converted to physical distances as

$$D_{\mathrm{real}}=D_{\mathrm{pixel}}\times \frac{10}{1680-1136}$$

where D_real_ is the distance in centimeters, and D_pixel_ is the distance in the aligned image-coordinate system. The reconstruction followed general principles of video-based kinematic measurement [33].

Formal intrinsic camera calibration and lens-distortion correction were not performed. After landmark-based coordinate alignment and ruler-based scale conversion, the two available estimates of each spatial coordinate were averaged to obtain a single coordinate value for each frame: x from Cameras 1 and 3, y from Cameras 2 and 3, and z from Cameras 1 and 2. The resulting frame-specific x-, y-, and z-coordinates were used to construct each flight trajectory.

A sphere 3 cm in diameter was rolled beside the rulers and processed using the same reconstruction procedure as the honey bee trajectories. Its recovered diameter and displacement relative to the ruler graduations were used as an overall check of spatial scale and reconstruction consistency. This sphere-based check did not constitute formal camera calibration and did not provide a trajectory-specific estimate of digitizing or reconstruction uncertainty. The same reconstruction procedure was applied consistently to all honey bee trajectories.

### 2.2. Data Analysis

The tracking procedure was implemented in Python 3.8 using the opencv-contrib-python library (version 4.5.5.62). The final honey bee trajectories used in the present analyses were extracted using the CSRT (Channel and Spatial Reliability Tracker) [34]. For each camera view, the focal honey bee was manually initialized by drawing a bounding box using cv2.selectROI(), a built-in function. Tracking then proceeded frame by frame using the built-in default parameters of CSRT without additional manual tuning. The centroid of each tracked bounding box was used as the honey bee’s two-dimensional image-plane position.

**Figure 2:**
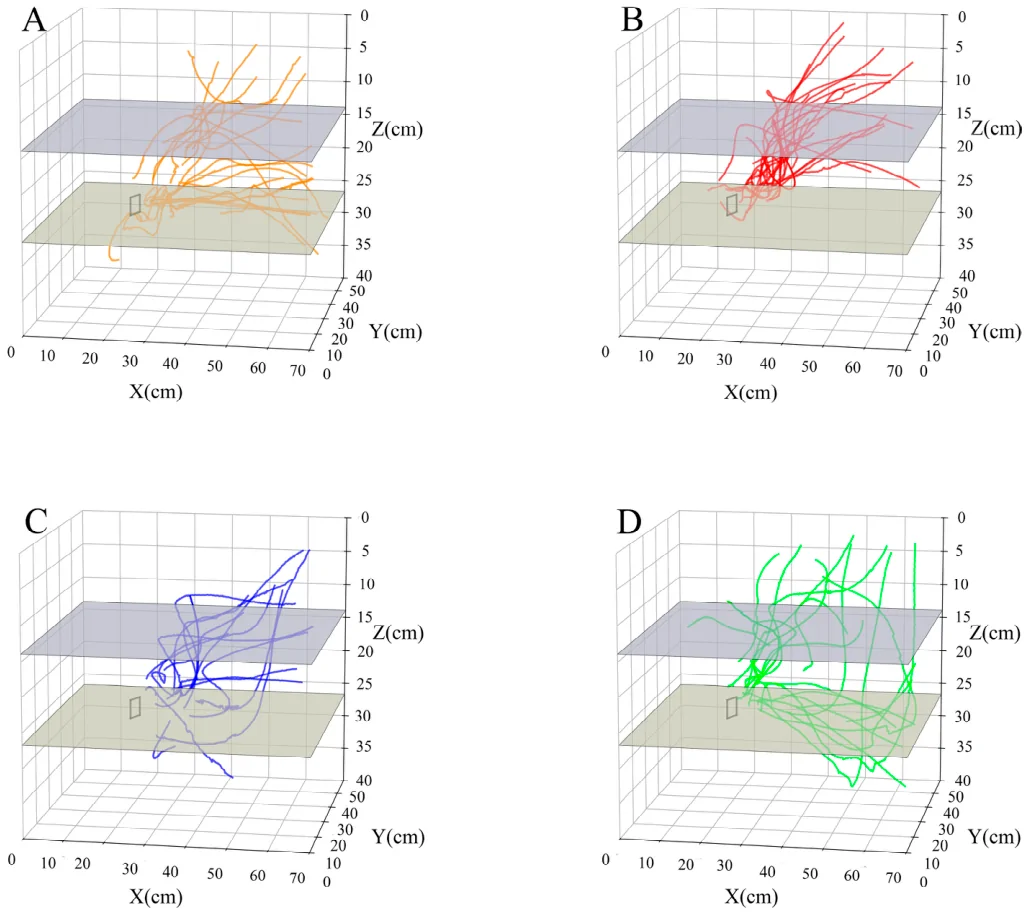
Three-dimensional landing trajectories of each group: (**A**) control, (**B**) 20 mg, (**C**) 40 mg, and (**D**) 60 mg. Each colored line represents the flight trajectory of an individual honey bee, and the black rectangle indicates the hive entrance. The translucent planes delimit the vertical spatial zones.

Following automatic tracking, the tracked positions were visually checked against the original image sequences and manually corrected whenever the tracked position did not correspond to the focal honey bee. Only the checked coordinate series were used for subsequent trajectory reconstruction and analysis. The reconstructed x-, y-, and z-coordinate series were plotted to show each honey bee’s approach near the hive entrance (Figure 2). Each honey bee contributed one complete landing trajectory. For movement calculations, the first position in each set of five consecutive frames was retained. At 500 frames s^−1^, the resulting sampling interval was 0.01 s. Local path bending was quantified using a discrete curvature estimate calculated from three successive retained three-dimensional positions, following Chen and Wu [35]. For retained positions *P_i_*_−1_, *P_i_*, and *P_i_*_+1_, the displacement vectors were defined as

*r_i_* = *P_i_* − *P_i_*_−1_ and *r_i_*_+1_ = *P_i_*_+1_ − *P_i_*

The angle between the two displacement vectors was calculated as

$$\theta_{i}=\arccos(\frac{r_{i}\cdot r_{i+1}}{\left\|r_{i}\|\|r_{i+1}\right\|})$$

and the discrete curvature estimate was calculated as

$$\kappa_{i}=\frac{\theta_{i}}{\left\|r_{i}\|+\|r_{i+1}\right\|}$$

Because the spatial coordinates were expressed in centimeters, *κ_i_* was expressed in cm^−1^. The same calculation was applied to all sets of three successive retained positions and to all trajectories. For brevity, this discrete estimate is termed “curvature” below. Because this discrete curvature estimate was calculated from three consecutive retained points, it may be sensitive to the temporal sampling interval and coordinate noise. It describes the magnitude of local path bending. Flight velocity was calculated from displacement between successive retained positions. For each honey bee, the 20 sampled positions with the highest curvature estimates were identified to characterize the most strongly curved local portions of that trajectory. Similar trajectory-based approaches have been used in animal-flight studies, in which extreme trajectory values, maximum curvature, or minimum radius of curvature was used to characterize the most strongly curved or tightest portions of flight trajectories [36,37,38]. These selected positions represent local kinematic features within one recorded trajectory rather than separate landing trials or separate flight trajectories.

Data were organized in Microsoft Excel 2021, analyzed in IBM SPSS Statistics 26, and plotted in GraphPad Prism 10.1.2. Depending on the variable, summaries are shown as medians with 95% confidence intervals or as violin plots with the distribution, median, and interquartile range. Between-treatment comparisons were evaluated with the Kruskal–Wallis H test, followed, when appropriate, by Bonferroni-adjusted pairwise comparisons. The within-treatment comparisons among the upper, middle, and lower zones were presented descriptively because the zone measurements originated from different portions of the same trajectories. Tests were two-sided, and an adjusted *p* value < 0.05 was considered statistically significant where inferential tests were applied.

**Figure 3:**
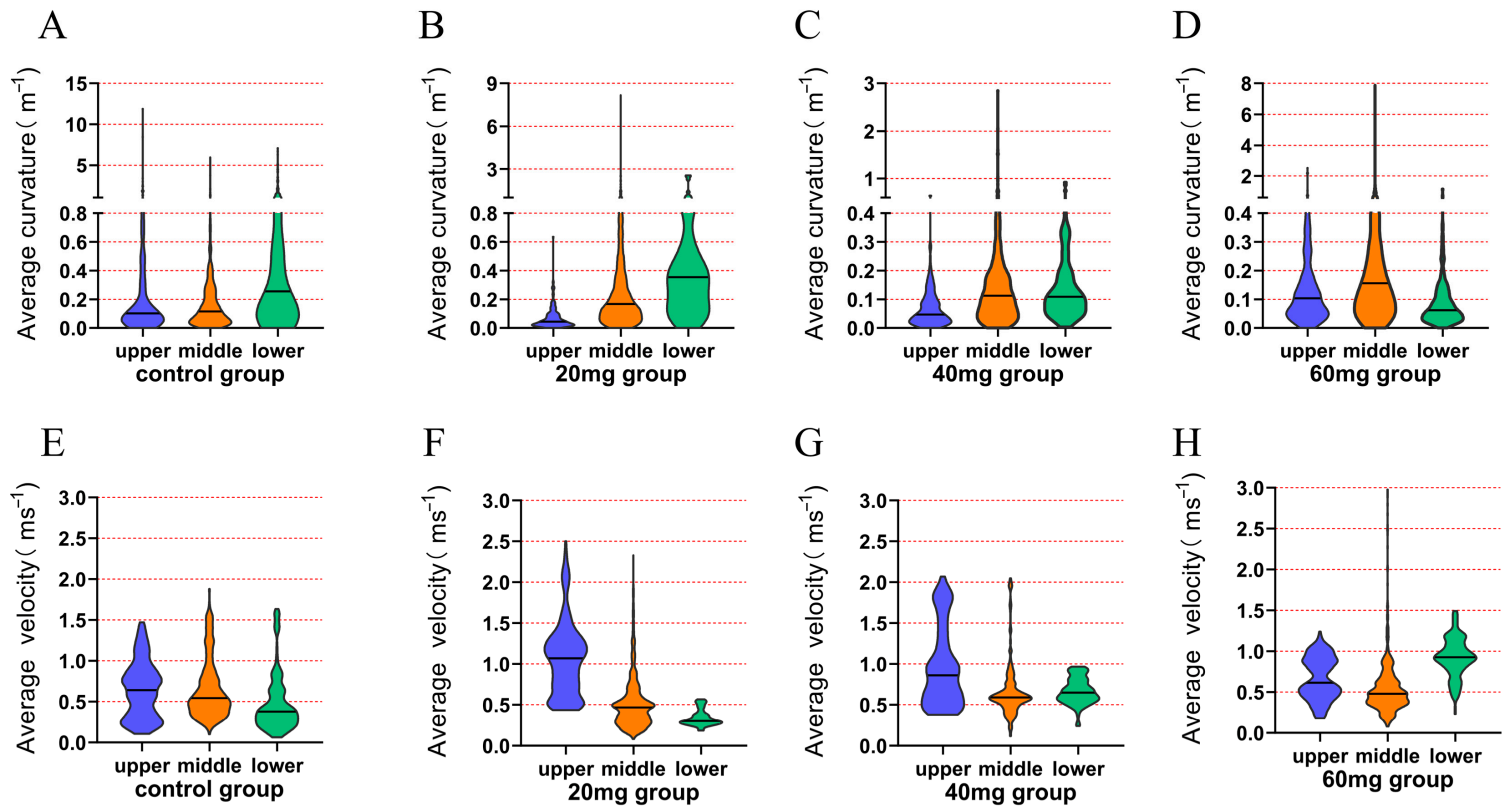
Trajectory discrete curvature estimates and flight velocity in the upper, middle, and lower spatial zones (control, *n* = 21; 20 mg, *n* = 22; 40 mg, *n* = 16; 60 mg, *n* = 21). Curvature estimates are shown for (**A**) control, (**B**) 20 mg, (**C**) 40 mg, and (**D**) 60 mg honey bees; corresponding flight velocity is shown in (**E**–**H**). Because values from the upper, middle, and lower zones originated from different portions of the same trajectories, the within-treatment zone patterns are presented descriptively without formal between-zone hypothesis testing.

Each honey bee was recorded during a single post-treatment landing approach and contributed one complete trajectory to the dataset. The successive frame-level positions were used to characterize the kinematics of that single recorded flight rather than repeated flight trials by the same individual. The 20 highest-curvature positions represented local features within a single trajectory, rather than separate landing events. Measurements from the upper, middle, and lower zones likewise originated from different portions of the same trajectory. Because the zone comparisons in Figure 3 therefore represented within-trajectory spatial variation, these patterns are presented descriptively without formal between-zone hypothesis testing.

The original treatment-level analyses did not include colony or sampling block as factors because complete block-level records were not retained. Accordingly, the statistical inference is restricted to comparisons among load treatments within the available set of complete trajectories, and the inability to account retrospectively for colony and sampling block is acknowledged as a limitation.

A trajectory was included in the final analysis only when the marked honey bee returned during the recording period and completed a normal landing approach, and a complete video record suitable for trajectory reconstruction was retained. For the purposes of the present analysis, a landing trajectory was defined as a complete and continuous flight approach within the recording volume; honey bees that contacted the ground and subsequently crawled toward the entrance were not included in the trajectory dataset. Honey bees that did not return, were not successfully recorded during camera operation, showed prolonged wandering rather than a definable landing approach, or otherwise did not provide a complete normal landing trajectory were not included in the final dataset. The original field records did not retain treatment-specific counts for excluded honey bees or separate counts for each exclusion category; these numbers therefore cannot be reconstructed retrospectively. The final dataset comprised 80 complete trajectories from 80 individual honey bees: control, *n* = 21; 20 mg, *n* = 22; 40 mg, *n* = 16; and 60 mg, *n* = 21. The final dataset therefore comprised one recorded landing trajectory from each of 80 individual honey bees. The trajectory data are provided in Supplementary Tables S1–S5, and the independent body-mass reference data are provided in Supplementary Table S6.

**Figure 4:**
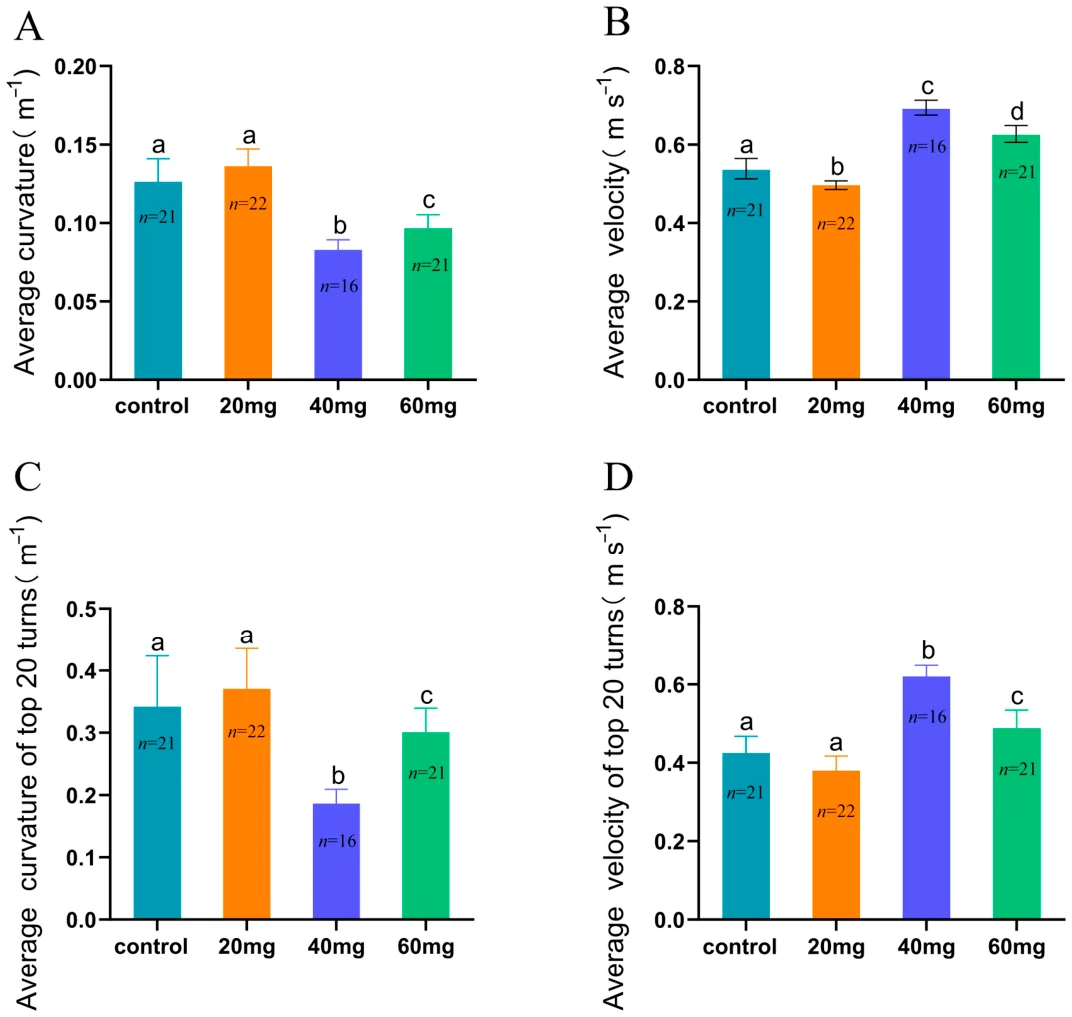
Trajectory discrete curvature estimates and flight velocity across load treatments (control, *n* = 21; 20 mg, *n* = 22; 40 mg, *n* = 16; 60 mg, *n* = 21). (**A**) Average full-trajectory curvature estimates. (**B**) Average full-trajectory flight velocity. (**C**) Average of the 20 highest local curvature estimates per honey bee. (**D**) Average flight velocity at the corresponding 20 sampled positions. Numbers within bars indicate the numbers of honey bees. Different lowercase letters indicate significant Bonferroni-adjusted pairwise differences following the Kruskal–Wallis test (adjusted *p* < 0.05); groups sharing a letter did not differ significantly.

**Figure 5:**
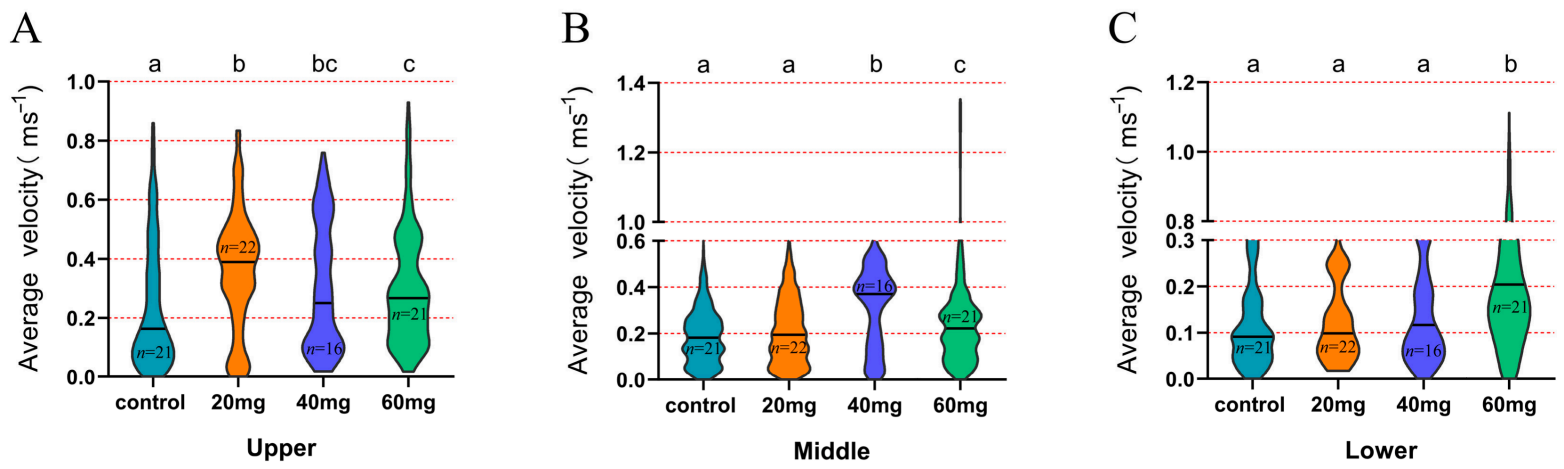
Vertical landing velocity by spatial zone: (**A**) upper, (**B**) middle, and (**C**) lower. Different lowercase letters indicate significant Bonferroni-adjusted pairwise differences following the Kruskal–Wallis test (adjusted *p* < 0.05); groups sharing a letter did not differ significantly. Numbers within violin plots indicate the numbers of honey bees.

In Figure 4 and Figure 5, different lowercase letters denote significant Bonferroni-adjusted pairwise differences after the Kruskal–Wallis test (adjusted *p* < 0.05). Groups sharing a letter did not differ significantly (adjusted *p* ≥ 0.05).

## 3. Results

### 3.1. Overall Flight Velocity and Trajectory Curvature

Average flight velocity was significantly higher, and average curvature estimate significantly lower, in the 40 and 60 mg groups than in the control and 20 mg groups (*p* < 0.05, Kruskal–Wallis test; Figure 4A,B). The 40 mg group also had higher average velocity and lower average curvature than the 60 mg group. Curvature did not differ significantly between the control and 20 mg groups, whereas average velocity was higher in the control group (*p* < 0.05; Figure 4A,B).

We also compared the 20 sampled positions with the highest curvature in each trajectory and the velocities recorded at those positions. Curvature at these positions was lower, and velocity higher, in the 40 and 60 mg groups than in the control and 20 mg groups (*p* < 0.05, Kruskal–Wallis test; Figure 4C,D). No significant difference was detected between the control and 20 mg groups. Compared with the 40 mg group, the 60 mg group had lower velocity and higher curvature at these sampled positions (*p* < 0.05; Figure 4C,D).

### 3.2. Variation in Flight Velocity and Curvature Among Vertical Zones

To descriptively compare different parts of the landing approach, we divided the recording volume along the vertical (z) axis into upper, middle, and lower zones. The lower zone was closest to the ground. Because measurements from these zones originated from different portions of the same trajectories, the zone-based patterns are presented descriptively without formal between-zone hypothesis testing.

Average curvature increased from the upper to the lower zone in the control and 20 mg groups (Figure 3A,B). Curvature also increased across zones in the 40 mg group, although the change was less pronounced (Figure 3C). In the 60 mg group, curvature increased from the upper to the middle zone and then decreased in the lower zone (Figure 3D). Velocity generally changed in the opposite direction. It decreased from the upper to the lower zone in the control and 20 mg groups (Figure 3E,F). Velocity also decreased in the 40 mg group, with relatively similar values in the middle and lower zones (Figure 3G). In the 60 mg group, velocity decreased from the upper to the middle zone and then increased in the lower zone (Figure 3H).

### 3.3. Vertical Velocity Among Load Treatments and Recording Zones

We compared the vertical velocities under different load treatments within each recording zone. In the upper zone, the 20 mg and 40 mg groups had significantly higher vertical landing velocity than the control, whereas the difference between the 60 mg group and the control was not significant (Figure 5A). In the middle zone, the vertical landing velocity of the 20 mg group decreased to the control level, while the 40 mg and 60 mg groups remained significantly higher than the control (*p* < 0.05, Kruskal–Wallis test; Figure 5B). In the lower zone, vertical landing velocity was significantly higher in the 40 mg and 60 mg groups than in the control and 20 mg groups (*p* < 0.05, Kruskal–Wallis test; Figure 5C).

### 3.4. Body Mass of the Independent Reference Sample and Relative Load Magnitude

In the independent body-mass reference sample, the mean body mass after removal of the hind-leg pollen pellets was 77.86 ± 4.28 mg (mean ± SD; range, 70.90–87.30 mg; *n* = 60). Colony-specific means were 77.69, 77.86, and 78.02 mg (*n* = 20 per colony). Relative to the overall reference-sample mean, the 20, 40, and 60 mg tags represented approximately 25.69%, 51.37% and 77.06%, respectively. These percentages provide descriptive population-level context for the magnitude of the experimental treatments and do not represent the actual relative loads of the individual honey bees used in the trajectory experiment. The individual measurements and descriptive statistics are reported in Supplementary Table S6.

## 4. Discussion

External thoracic load was associated with differences in the landing paths of returning foragers. Across the complete landing approach, control and 20 mg honey bees generally combined lower velocity with greater curvature, whereas the 40 and 60 mg groups showed higher velocity and lower curvature. The pattern was not a progressive response to increasing load: the 40 mg group had the highest overall velocity and the lowest overall curvature, while the 60 mg group changed differently across the vertical zones, particularly near the ground. Thus, treatment differences depended on load level and position within the landing approach.

Curvature estimates were broadly similar between the control and 20 mg groups during the recorded approach. Honey bees can alter aerodynamic force production through changes in stroke amplitude and other wing kinematics, while added load increases both mechanical and metabolic demands [2,3,4]. In this context, the broadly similar trajectory characteristics of the control and 20 mg groups indicate that the 20 mg tag group responded similarly to controls under the present experimental conditions. Because individual flight capacity, wing kinematics, aerodynamic forces, and physiological responses were not measured, the trajectories alone cannot determine how this load was accommodated.

Honey bees carrying 40 mg followed faster and less curved trajectories than the control and 20 mg groups. Greater mass increases momentum, which may make rapid deceleration combined with tight turning more demanding. Comparative work in bumblebees likewise shows that load magnitude and position alter the balance between stability and maneuverability, and that speed and path sinuosity contribute jointly to performance in clutter [11,12]. Thus, the faster and straighter trajectories of the 40 mg group may reflect the combined effects of additional mass, momentum, and the available capacity for flight-force production. However, the present measurements do not distinguish a direct mechanical consequence of loading from active modulation of the approach trajectory.

The 60 mg treatment differed most clearly from the lower-load groups. Among the 21 complete normal landing trajectories included in the analysis, curvature was relatively high in the upper and middle zones but decreased in the lower zone, where velocity increased. The largest tag was therefore associated with different trajectory characteristics at successive parts of the analyzed approach, rather than with one uniform change along the entire path. During the field experiment, approximately 25% of the observed honey bees carrying the 60 mg tag did not land directly at the hive entrance but instead contacted the ground shortly before reaching the entrance and then crawled a short distance toward the hive. These honey bees were not included in the final trajectory dataset. The exact ground-contact positions and distances from the hive entrance were not systematically measured and therefore could not be quantified reliably. Accelerated landing has been reported in a stingless bee [39], but species, entrance structure, sensory conditions, and ecological context differ. In the 60 mg group, velocity and curvature changed differently among the upper, middle, and lower recording zones, indicating that the trajectory pattern varied across successive portions of the approach. One possible explanation is that the added thoracic load altered the mechanical demands associated with deceleration and turning near the entrance. However, because flight forces and wing kinematics were not measured, the present data cannot determine whether this pattern resulted from a direct mechanical effect of the tag, active modulation of flight control, or a combination of these factors. The 60 mg result is therefore best described as a position-dependent difference in the recorded approach rather than evidence for a defined landing strategy or mechanism.

The analysis of the 20 positions with the highest curvature estimates provides additional information about the most strongly curved portions of each trajectory. The use of local curvature extrema to characterize strongly curved portions of animal flight trajectories is consistent with previous studies of bat maneuvering flight [36,37,38]. These positions should not be interpreted as 20 independent turns because adjacent sampled positions may belong to the same continuous trajectory segment. Their value lies in showing whether the extreme curved portions of the recorded paths differed among load treatments.

Vertical velocity also varied over the approach. Control and 20 mg honey bees generally slowed near the entrance, consistent with the role of visual-motion information in regulating approach speed [18,40]. The 40 mg group retained higher vertical velocity while completing the recorded approach, and the 60 mg group remained relatively fast through much of the observation volume. The timing and magnitude of deceleration may therefore have differed among treatments. Wind, turbulence, and visual structure can also alter speed, height control, and stability [28,41,42]. The present data establish load-associated trajectory differences but do not identify a single sensory or aerodynamic process responsible for them.

The independent reference sample provides descriptive population-level context for the magnitude of the fixed loads. After removal of the hind-leg pollen pellets, body mass averaged 77.86 ± 4.28 mg (mean ± SD; *n* = 60). Relative to this reference-sample mean, the 20, 40, and 60 mg tags corresponded to approximately 25.69%, 51.37%, and 77.06%, respectively (Supplementary Table S6). These percentages should not be interpreted as the actual relative loads of the individual honey bees used in the trajectory experiment because individual body mass was not measured in those honey bees. Thus, the reference-sample values provide only approximate population-level context for the magnitude of the experimental treatments. The 20 mg group remained broadly similar to controls, whereas the 40 and 60 mg groups showed more pronounced and non-monotonic trajectory differences. The observed non-monotonic pattern suggests the testable hypothesis that increasing thoracic load may alter the balance between maintaining forward velocity and trajectory adjustment during the final landing approach. Under this hypothesis, intermediate and higher loads could produce different combinations of velocity and path curvature rather than a simple progressive response to increasing load. This hypothesis could be tested by simultaneously measuring three-dimensional trajectories, wing kinematics, and aerodynamic forces across load levels.

An additional consideration is that the experimental loads used here were fixed lead tags attached externally to the dorsal thorax and should not be regarded as direct equivalents of natural foraging loads. Nectar is carried internally in the crop, whereas pollen is carried externally on the hind legs. These loading configurations differ from a dorsal thoracic tag in load position and distribution and may therefore have different effects on the center of mass, moment of inertia, and aerodynamic profile of the honey bee. Moreover, because wing kinematics and aerodynamic forces were not measured, possible effects of the tags on local airflow, aerodynamic drag, or wing movement cannot be excluded. The observed differences should therefore be interpreted as responses associated with the combined experimental condition of externally attached thoracic tags, including their mass, position, and physical configuration, rather than as effects of added mass alone or as direct evidence of the effects of naturally carried nectar or pollen. In addition, the present findings are specific to *Apis mellifera*, and their applicability to other bee species remains to be determined.

Taken together, the differences in velocity, curvature, and vertical movement show that external load was associated with both path geometry and the progression of the approach. These trajectory measurements do not, however, directly identify changes in maneuvering ability, individual flight-performance capacity, or the flight-control mechanisms responsible for the observed patterns. Accelerated or crash-like approaches have also been reported at stingless bee nest entrances, although those observations involve different species and entrance structures [39,43]. An important limitation of the present study concerns the available sampling and disposition records. Although complete video records were retained for all 80 honey bees included in the trajectory analyses, the original field records did not retain the exact number of sampling blocks completed for each colony, the treatment-specific numbers of honey bees initially assigned, or treatment-specific counts and video records for honey bees that did not contribute a complete normal landing trajectory. Consequently, treatment-specific attrition and possible effects of load on return probability or successful completion of a normal landing approach cannot be evaluated retrospectively. The smaller number of retained trajectories in the 40 mg group (*n* = 16) may also have reduced statistical power for this treatment. Complete colony- and block-level information was also unavailable for adjustment for colony or sampling block in the statistical analysis. Although adhesive-based thoracic tagging and color marking are widely used in honey bee behavioral studies [29,30,31,32], their independent effects were not specifically evaluated in the present experiment. Control honey bees received the same color marking and a sham application of adhesive, so these procedures were not unique to the load treatments. Nevertheless, small independent effects of the adhesive or marking color cannot be completely excluded. The present inference is therefore restricted to trajectory characteristics of the successfully recorded honey bees and should not be extended to treatment effects on return or landing success. Future studies using similar block-based field designs would benefit from prospectively retaining complete block-level allocation and disposition records for all experimental individuals.

A further methodological consideration concerns the three-camera trajectory reconstruction. Temporal correspondence among camera views was established using the recording-alignment procedure described above. However, because no independent timing marker was recorded, residual inter-camera timing offsets were not independently quantified. This may introduce uncertainty into frame-wise positional and derived kinematic estimates, including velocity and curvature. For spatial reconstruction, complementary camera views, fixed hive landmarks, and ruler-derived scaling were combined. Formal intrinsic camera calibration and lens-distortion correction were not performed. The 3-cm sphere provided an overall check of spatial scale and reconstruction consistency, but it does not represent a trajectory-specific error estimate. These limitations should be considered when interpreting absolute positional and kinematic values. Because all treatment groups were recorded and processed using the same procedure, the reconstructed trajectories are most appropriately interpreted in a comparative framework, whereas absolute kinematic values should be interpreted with caution. Future work could incorporate independent synchronization signals, checkerboard-based camera calibration and lens-distortion correction, and repeated reconstructions to quantify trajectory-specific uncertainty.

Future experiments could combine three-dimensional trajectory recording with measurements of individual body mass, crop load, wing kinematics, body orientation, and aerodynamic force. Recording entrance traffic, wind, temperature, visual conditions, and starting position would further clarify how external load interacts with the physical and social environment during landing. Such measurements could help distinguish the direct mechanical effects of added mass from active modulation of the approach. The three-dimensional records of individual foragers show that fixed external loads were associated with distinct landing trajectories at a natural hive entrance. The present results may also offer comparative information for studies of small flying systems approaching restricted landing sites while carrying variable payloads.

## 5. Conclusions

Honey bee foragers carrying fixed, externally attached thoracic lead tags exhibited distinct three-dimensional landing-approach trajectories near the hive entrance. Honey bees in control and 20 mg groups generally approached at lower velocities along more curved paths, whereas honey bees carrying the 40 mg tag followed faster and straighter trajectories. In the 60 mg group, velocity and curvature varied among the upper, middle, and lower recording zones. These findings show that recorded trajectory characteristics differed among the fixed thoracic lead-tag treatments rather than following a simple progressive response to increasing tag mass. Because individual flight capacity, wing kinematics, and aerodynamic forces were not measured, the observed trajectory differences do not directly demonstrate changes in maneuvering ability or identify a specific mechanical mechanism or landing strategy. Because the experimental lead tags differed from natural nectar and pollen loads in their position, distribution, and physical configuration, the present findings should not be interpreted as directly quantifying the effects of natural foraging loads. The present findings are specific to *Apis mellifera* carrying fixed externally attached thoracic lead tags during the short-range final landing approach following release approximately 10 m from the hive. These experimental conditions should be considered when extrapolating the results to other loading situations or longer-range return flights. Overall, these field measurements demonstrate differences in the final landing approach among honey bees carrying different fixed thoracic lead tags and provide a basis for further investigations combining trajectory, individual body-mass, wing-kinematic, and aerodynamic-force measurements.

## Data Availability

The original contributions presented in this study are included in the article/Supplementary Materials. Further inquiries can be directed to the corresponding author.

## Supplementary Materials

The following supporting information can be downloaded at: https://www.mdpi.com/article/10.3390/ani16193152/s1 for Tables S1–S7 and https://doi.org/10.5281/zenodo.22976003 for Supplementary Videos S1–S4. Table S1: Raw flight data of each bee; Table S2: Raw data of discrete curvature estimates and speed of each group; Table S3: Raw data of top 20 discrete curvature estimates and speed of each group; Table S4: Raw data of discrete curvature estimates and speed of three spaces along the *z*-axis (upper, middle and lower spaces) of each group; Table S5: Raw data of the vertical landing velocities (upper, middle and lower spaces) of each group; Table S6: Body masses of an independent reference sample of returning honey bee foragers and population-level estimates of the relative magnitude of the fixed external loads; Table S7: Meteorological conditions during the observation periods on experimental days; Video S1: Representative landing approach of a control honey bee marked with white paint; Video S2: Representative landing approach of a honey bee marked with red paint and carrying a 20 mg thoracic lead tag; Video S3: Representative landing approach of a honey bee marked with blue paint and carrying a 40 mg thoracic lead tag; Video S4: Representative landing approach of a honey bee marked with green paint and carrying a 60 mg thoracic lead tag.
